# A 10 Year Long‐Lived Cellular and Humoral MERS‐CoV Immunity Cross‐Recognizing the Wild‐Type and Variants of SARS‐CoV‐2: A Potential One‐Way MERS‐CoV Cross‐Protection Toward a Pan‐Coronavirus Vaccine

**DOI:** 10.1002/jmv.70071

**Published:** 2025-01-17

**Authors:** Bandar Alosaimi, Maaweya Awadalla, Wael Alturaiki, Zhao Chen, Zhaoyong Zhang, Airu Zhu, Fatimah Rebh, Abeer N. Alshukairi, Jincun Zhao, Haitham S. Alkadi

**Affiliations:** ^1^ Research Center, King Fahad Medical City, Riyadh Second Health Cluster Riyadh Saudi Arabia; ^2^ Department of Medical Laboratory Sciences College of Applied Medical Sciences, Majmaah University, Majmaah Riyadh Region Saudi Arabia; ^3^ State Key Laboratory of Respiratory Disease, Guangzhou Institute of Respiratory Disease, The First Affiliated Hospital of Guangzhou Medical University Guangzhou Guangdong China; ^4^ Department of Internal Medicine Section of Infectious Diseases, Prince Mohammed Bin Abdulaziz Hospital Riyadh Saudi Arabia; ^5^ Department of Medicine King Faisal Specialist Hospital and Research Center Jeddah Saudi Arabia; ^6^ Guangzhou laboratory, Bio‐island Guangzhou Guangdong China

**Keywords:** BA.5 variant, cellular immunity, cross‐reactivity, Delta variant, humoral immunity, MERS‐CoV, SARS‐CoV‐2, short‐term T cell line

## Abstract

MERS is a respiratory disease caused by MERS‐CoV. Multiple outbreaks have been reported, and the virus co‐circulates with SARS‐CoV‐2. The long‐term (> 6 years) cellular and humoral immune responses to MERS‐CoV and their potential cross‐reactivity to SARS‐CoV‐2 and its variants are unknown. We comprehensively investigated long‐lasting MERS‐CoV‐specific cellular and humoral immunity, and its cross‐reactivity against SARS‐CoV‐2 and its variants, in individuals recovered from MERS‐CoV infection 1–10 years prior. Two cohorts of MERS‐CoV survivors (31 unvaccinated, 38 COVID‐19 vaccinated) were assessed for MERS‐CoV IgG, memory CD4^+^/CD8^+^ T cells, and neutralizing antibodies against MERS‐CoV and SARS‐CoV‐2 variants. MERS‐CoV IgG levels and T cell responses were higher in the 1–5 vs 6–10 year postinfection groups. Vaccinated MERS‐CoV survivors had significantly elevated MERS‐CoV IgG and neutralization compared to unvaccinated. Both groups demonstrated cross‐reactive neutralization of SARS‐CoV‐2 variants. MERS‐CoV survivors vaccinated against SARS‐CoV‐2 had higher anti‐MERS IgG, cellular immunity, and neutralization than unvaccinated survivors. MERS‐CoV immune responses can persist for a decade. COVID‐19 vaccination boosted humoral and cellular immunity in MERS‐CoV survivors, suggesting the benefits of vaccination for this population. These findings have implications for pan‐coronavirus vaccine development.

## 1 Introduction

Middle East Respiratory Syndrome Coronavirus (MERS‐CoV) is a betacoronavirus that was first identified in 2012 after causing severe acute respiratory illness in Saudi Arabia. Since the first report of MERS‐CoV in 2012, a total of 2600 confirmed cases with 935 associated deaths have been reported globally, resulting in an overall case‐fatality rate of 35.5% [1, 2]. The majority of these MERS‐CoV cases (2193 cases, 84%) and associated deaths (854 deaths) have been reported from the Kingdom of Saudi Arabia [1]. Clinical manifestations of the disease include fever, cough, shortness of breath, and gastrointestinal symptoms. Other symptoms may involve myalgia, fatigue, and sore throat. In severe cases, MERS‐CoV can lead to pneumonia, acute respiratory distress syndrome (ARDS), and multiorgan failure [3]. The clinical course can progress rapidly, often requiring hospitalization and intensive care [1, 3]. Upon infection, the host's innate immune system recognizes MERS‐CoV through cytosolic and endosomal RNA sensors. Retinoic acid‐inducible gene I (RIG‐I) and toll‐like receptors (TLRs), specifically TLR2, TLR3, and TLR7, play key roles in the recognition of viral RNA, leading to the production of type I interferons and pro‐inflammatory cytokines [4]. MERS‐CoV infections induced the expression of several cytokines, including TNF‐α, IL‐6 IL‐12 IFN‐γ, as well as chemokines such as MCP‐1/CCL‐2, RANTES/CCL‐5, MIP‐1α/CCL‐3, IP‐10/CXCL‐10, and IL‐8 in human macrophages [4]. The production of these inflammatory mediators may play a significant role in the pathogenesis of MERS‐CoV infection [4, 5, 6]. MERS‐CoV infection shows markedly high levels of complement proteins C3a and C5a, factor P, IL‐8, and RANTES, leading to disease severity, acute respiratory distress syndrome (ARDS), and higher mortality rates [7]. MERS‐CoV induces T‐cell death through both extrinsic and intrinsic pathways [4]. MERS‐CoV infections also trigger T helper 17 (Th17) cytokines, which activate TNF‐α, IL‐1, IL‐6, IL‐8, and MCP‐1 [4, 5, 6, 8]. Additionally, MERS‐CoV infection decreases genes that encode Th1 and Th2 cytokines and chemokines in the lower respiratory tract [9].

Variants of MERS‐CoV can arise due to mutations, which may impact transmissibility, immune evasion, and virulence. MERS‐CoV exhibits genetic diversity, particularly among strains isolated from camels and humans [10]. Genomes obtained directly from MERS patients revealed three distinct MERS‐CoV genotypes [10, 11]. Analysis of the MERS‐CoV genomes demonstrated the anticipated accumulation of genetic diversity, including mutations in the spike (S) protein [10]. These mutations can influence receptor binding, replication efficiency, and sensitivity to neutralizing antibodies.

Coronaviruses that infect humans are classified into two genera: alpha coronaviruses and beta coronaviruses [12]. Coronaviruses are classified into different genera, with alpha and beta coronaviruses being the most notable. The endemic coronaviruses responsible for the common cold, primarily HCoV‐229E and HCoV‐NL63, belong to the alpha coronavirus genus, while HCoV‐OC43 and HCoV‐HKU1 are classified as beta coronaviruses. Among the beta coronaviruses, the most significant members include SARS‐CoV‐1, SARS‐CoV‐2, and MERS‐CoV [13, 14, 15]. Phylogenetic analyses reveal that SARS‐CoV‐2 shares approximately 76% genomic homology with SARS‐CoV‐1, while MERS‐CoV exhibits about 50% homology with both SARS strains. Notably, the Spike (S) protein shows about 77% sequence similarity between SARS‐CoV‐1 and SARS‐CoV‐2, and approximately 32% similarity with MERS‐CoV. This close genetic relationship suggests a potential for cross‐reactivity in immune responses, particularly between the two SARS strains [12, 16, 17, 18]. Previous study showed that individuals infected with MERS‐CoV showed long‐lasting antibody and T‐cell and B‐cell responses, up to 6.9 years after the initial infection [2]. Long‐term immune responses in individuals recovered from SARS‐CoV‐1 infection have been reported.

Previous studies have demonstrated that even 11–12 years postinfection, SARS‐CoV‐1 survivors showed robust T‐cell responses, characterized by memory T cells that can effectively respond to viral antigens. Additionally, while antibody levels decline over time, neutralizing antibodies specific to SARS‐CoV‐1 can still be detected in many recovered patients, indicating a lasting immune imprint [19, 20, 21, 22].

However, there have been no studies showing long‐lasting immunity specific to MERS‐CoV and the potential SARS‐CoV2 cross‐reactive beyond 6.9 years postinfection. Our study examined the long‐lasting immunity specific to MERS‐CoV and the potential Wild‐type and variants of SARS‐CoV‐2 cross‐reactivity.

## 2 Materials and Methods

### 2.1 Study Design, Human Subjects, and Sample Collection

The study was conducted between 2020 and 2023 at King Fahad Medical City in Riyadh, Saudi Arabia. A total of 539 individuals who had laboratory‐confirmed MERS‐CoV infection and survived were identified from the hospital database, of which 137 were diseased. The potential participants were contacted by phone and provided full information about the study's purpose and overview. Verbal consent was obtained from patients who agreed to participate. Those who gave verbal consent were asked to visit the study site to sign a consent form and provide samples. The telephone‐based invitation was conducted in three stages; those who did not respond were followed up with two calls each month for 6 months. 69 MERS‐CoV survivors (31 without and 38 with COVID‐19 vaccination) visited the study site and signed the informed consent form after meeting the eligibility criteria. MERS‐CoV‐recovered individuals were divided into two groups based on time since infection: 1–5 years postinfection (*n* = 10) and 6–10 years postinfection (*n* = 12). Individuals with influenza‐like illness, common cold symptoms, autoimmune diseases, inflammatory diseases, and malignancies were excluded. 10 mL of venous blood was collected using lithium heparin (BD Biosciences, San Jose, CA, USA). The participants were 70% male and 30% female, aged between 18 and 75 years, with a mean age of 47.18 ± 13.69 (median age 47) years. Healthy, non‐MERS‐CoV‐infected individuals served as a control group. We collected extensive clinical data on participants, including their SARS‐CoV‐2 infection history and serostatus. Notably, none of the participants had a SARS‐CoV‐2 infection, ensuring that our sample was free from biases related to prior SARS‐CoV‐2 infection, so we did not look at specific anti‐SARS‐CoV‐2 antibodies for all groups. The King Fahad Medical City Institutional Review Board (IRB) reviewed and approved the research protocol (IRB log number: 20‐703). The work was conducted in accordance with the principles of the Declaration of Helsinki, and participants' data were treated with utmost confidentiality. The demographic characteristics of the study participants are summarized in Table 1.

**Table 1:** Demographics characteristics of the study participants.

Variable	MERS Recovered Individuals (*n* = 70)	Healthy individuals (Non‐MERS‐CoV‐infected) (*n* = 18) (%)
Age, Y, mean ± SD (median)	47.18 ± 13.69 (47)	61 ± 20.4 (61.5)
< 50 year	40 (57%)	12 (66.7%)
≥ 51 year	30 (43%)	6 (33.3%)
Male, *n* (%)	49 (70%)	14 (77.8%)
Female, *n* (%)	21 (30%)	4 (22.2%)
HCW, *n* (%)	10 (14%)	—
COVID‐19 vaccine	38 (54.3%)	—
Non‐COVID‐19 vaccine	32 (45.7%)	18 (100%)
Hospital isolation for MERS‐CoV, *n* (%)	54 (77%)	—
Intensive care unit (ICU)	16 (23%)	—
Contact with MERS‐CoV infected patients, *n* (%)	33 (47%)	
Contact with camels, *n* (%)	12 (17%)	—
Chronic conditions:		
Heart disease, *n* (%)	5 (7%)	—
Diabetes (Type 2), *n* (%)	18 (25%)	—
Hypertension, *n* (%)	13 (18%)	—
Malignancy, *n* (%)	1 (1%)	—
Kidney disease, *n* (%)	1 (1%)	—
Asthma, *n* (%)	2 (5%)	—
Pre‐existing condition		
Kidney disease, *n* (%)	1 (1%)	—

*Note:* Data were expressed as mean, median, or number (%). 32 unvaccinated, 38 COVID‐19 vaccinated.

### 2.2 Peptide Libraries

A peptide library was created by synthesizing a set of 12‐20‐mer peptides that overlapped by 10 amino acids from the four structural components (S1, S2, N, and ME) of MERS‐CoV (EMC strain) and SARS‐CoV‐2 (WT strain), which comprised the N‐ and C‐terminal portions of the spike (S) glycoprotein, the nucleocapsid (N) protein, the transmembrane (M), and envelope (E) proteins. Individual peptide stocks were used to prepare working solutions in RPMI Medium 1640. All peptide stocks and working solutions were stored at −20°C. The mega peptide pool of SARS‐CoV‐2 and MERS‐CoV was used in this study for PBMC stimulation. Amino acid sequences of the synthetic peptides are shown in Supporting Information S1: Table 1.

### 2.3 PBMCs and Plasma Isolation

PBMCs were isolated from fresh blood collected in anticoagulant tubes using density gradient centrifugation with Ficoll‐Paque (GE Healthcare). Cell counting was performed using the Countess automated cell counter (Invitrogen), and viability was assessed using trypan blue staining. Isolated PBMCs were frozen in liquid nitrogen for 2 months. The isolated plasma was aliquoted and stored at −80°C.

### 2.4 Quantification of the MERS‐CoV IgG Antibody

Circulating plasma levels of MERS‐CoV‐specific IgG antibodies were quantified using the Middle East Respiratory Syndrome‐Coronavirus IgG (MERS IgG) ELISA kit from MyBioSource, following the manufacturer's instructions. The plate was measured by detecting the absorbance at 450 nm using the Multiskan FC Microplate Photometer (Thermo Scientific).

### 2.5 Detection of MERS‐CoV‐Specific IgG Antibody Cross‐Recognizing and Binding to SARS‐CoV‐2 Spike Proteins

We conducted a pilot reverse experiment using an ELISA plate pre‐coated with the SARS‐CoV‐2 spike protein (SARS‐CoV‐2 virus IgG antibody detection kit BGI, Shenzhen, China) to measure the binding of MERS‐IgG to the spike protein of SARS‐CoV‐2 following the manufacturer's instructions.

### 2.6 Inhibitory Effect of MERS‐CoV Specific IgG on Binding of SARS‐CoV‐2 Spike RBD With ACE2 Protein

We conducted a competitive ELISA assay using the SARS‐CoV‐2 (2019‐nCoV) inhibitor screening ELISA kit (Si‐no Biological from Beijing, China) following the manufacturer's instructions. The inhibition value was calculated using the following equation:

Inhibition(%)=(1−ODvalueofSample/ODvalueofNegativeControl)×100

### 2.7 Focus Reduction Neutralization Test (FRNT) of SARS‐CoV‐2 and MERS‐CoV

SARS‐CoV‐2 variants (WT, Delta, BA.5) and MERS‐CoV (HCoV‐EMC/2012, Nigeria/NV1657, ChinaGD01) were cultured at Guangzhou Customs District Technology Center BSL‐3 Lab. Plasma samples were diluted (1:20 ~ 1:81920) and mixed with 100–200 FFU of SARS‐CoV‐2 or MERS‐CoV at 37°C for an hour. Fifty microliters of the mix were added to 96‐well cell plates (Vero E6 for SARS‐CoV‐2; Vero 81 for MERS‐CoV). After an hour at 37°C, cells were covered with overlay media for 24 h (MEM with 1.6% carboxymethylcellulose for SARS‐CoV‐2, 0.8% for MERS‐CoV). Plates were treated with 4% paraformaldehyde, permeabilized with 0.2% Triton X‐100, and stained with anti‐SARS‐CoV/SARS‐CoV‐2 Nucleocapsid Rabbit PAb for SARS‐CoV‐2, and anti‐MERS‐CoV Nucleocapsid Rabbit PAb for MERS‐CoV. HRP‐conjugated goat anti‐rabbit IgG was used as the secondary antibody. Foci were visualized with KPL TrueBlue Peroxidase Substrate and read by CTL ImmunoSpot S6 Ultra reader for FRNT50 calculation.

### 2.8 Ex Vivo T‐Cell Response Assessment via IFN‐γ and TNF‐α ELISPOT

MERS‐CoV‐specific and SARS‐CoV‐2 reactive T‐cell responses were evaluated using the enzyme‐linked immunospot assay (ELISpot) using the human IFN‐γ and TNF‐α ELISPOT assay kit (U‐CyTech biosciences, Utrecht, The Netherlands) according to the manufacturer's instructions as previously described [13, 23].

PBMCs were stimulated with mega peptide pool of SARS‐CoV‐2 and MERS‐CoV (Final concentration 1 μg of each peptide/mL) containing structural proteins of four MERS‐CoV (EMC strain) and SARS‐CoV‐2 N‐ and C‐terminal portions of the spike (S1 and S2) glycoprotein, the nucleocapsid (N) protein, and the transmembrane with the envelope (ME) protein. The plates were analyzed and counted using an AID EliSpot Reader System (AID GmbH). The cutoff for the ELISPOT assay was determined based on the T‐SPOT manual recommendations [24]: a difference of at least 6 spots was applied when the negative control showed 5 or fewer spots, while a ratio of at least 2 was used when the negative control had 6 or more spots. The ELISPOT assay results are presented as spot‐forming cells (SFCs) per 1 × 10^6^ cells.

### 2.9 Intracellular Cytokine Staining Assay (ICS)

Recovered PBMCs were stimulated with peptide pools containing structural proteins of four MERS‐CoV (EMC strain) (final concentration 1 μg of each peptide/mL) or SARS‐CoV‐2 (WT strain) (final concentration 1 μg of each peptide/mL) N‐ and C‐terminal portions of the spike (S1 and S2) glycoprotein, the nucleocapsid (N) protein, the transmembrane with the envelope (ME) protein as described by [25, 26]. All flow cytometry data were acquired on a BD FACSVerse and analyzed using FlowJo software (Tree Star Inc.).

### 2.10 Short‐Term T Cell Line Development

TCLs were generated from PBMCs of MERS‐CoV survivors with and without COVID‐19 vaccination. PBMC viability was between 85% and 98% post‐liquid thawing. PBMCs thawed from liquid nitrogen were stimulated with peptide pools of MERS‐CoV or SARS‐CoV‐2, each at a concentration of 200 nM/peptide. The cells were cultured for 12 days with recombinant human IL‐2 (rhIL‐2) at a final concentration of 100 U/mL. Half of the medium was replaced every 3 days. After proliferation, rhIL‐2 was removed from the culture medium and the cells were rested overnight before stimulation. Subsequently, an ICS (intracellular cytokine staining) assay was performed on the TCLs using various peptide pools to measure specific and cross‐reactive T‐cell responses as previously described [25]. A series of intracellular cytokine (IFN‐γ and TNF‐α) staining assays were utilized to measure specific and cross‐reactive T cells, as previously described [25]. Briefly, the IL‐2‐free TCLs were washed with culture medium and stimulated with various peptide pools (200 nM) (S/N/M/from MERS‐CoV (EMC strain) or SARS‐CoV‐2 (WT stain)) for 6 h. Cells were fixed and permeabilized with Cytofix, and surface and intracellular markers were stained before the detection of flow cytometry. The following anti‐human monoclonal antibodies were used: BUV395‐CD3 (UCHT1; BD), BB515‐CD4 (RPA‐T4; BD), BV605‐CD8 (SK1; BD), APC‐IFN‐g (B27; BD), PE‐Cy7‐TNF (MAb11; Biolegend), and SB436‐CD19 (HIB19; Invitrogen).

### 2.11 Statistical Analysis

Statistical analysis was performed using GraphPad Prism 10 (GraphPad Software, California, USA). Continuous variables were described using median and upper and lower interquartile ranges (IQR). Categorical variables were expressed as means, medians, or percentages (%). Regarding the experiment results, we assessed whether the measurements were within the normal range. Non‐parametric tests were used if the data did not follow a normal distribution according to the Shapiro–Wilk normality test. The number of study participants (*n*) and *p* values for all experiments are shown in each figure. The Pearson *r* correlation coefficient analysis was also used. Statistical significance was defined as a *p*‐value less than 0.05. The sample sizes for each experiment varied due to the availability of sufficient sample volume for each specific assay.

## 3 Results

### 3.1 MERS‐CoV Specific Immunoglobulin G Circulating Over Time

**Figure 1:**
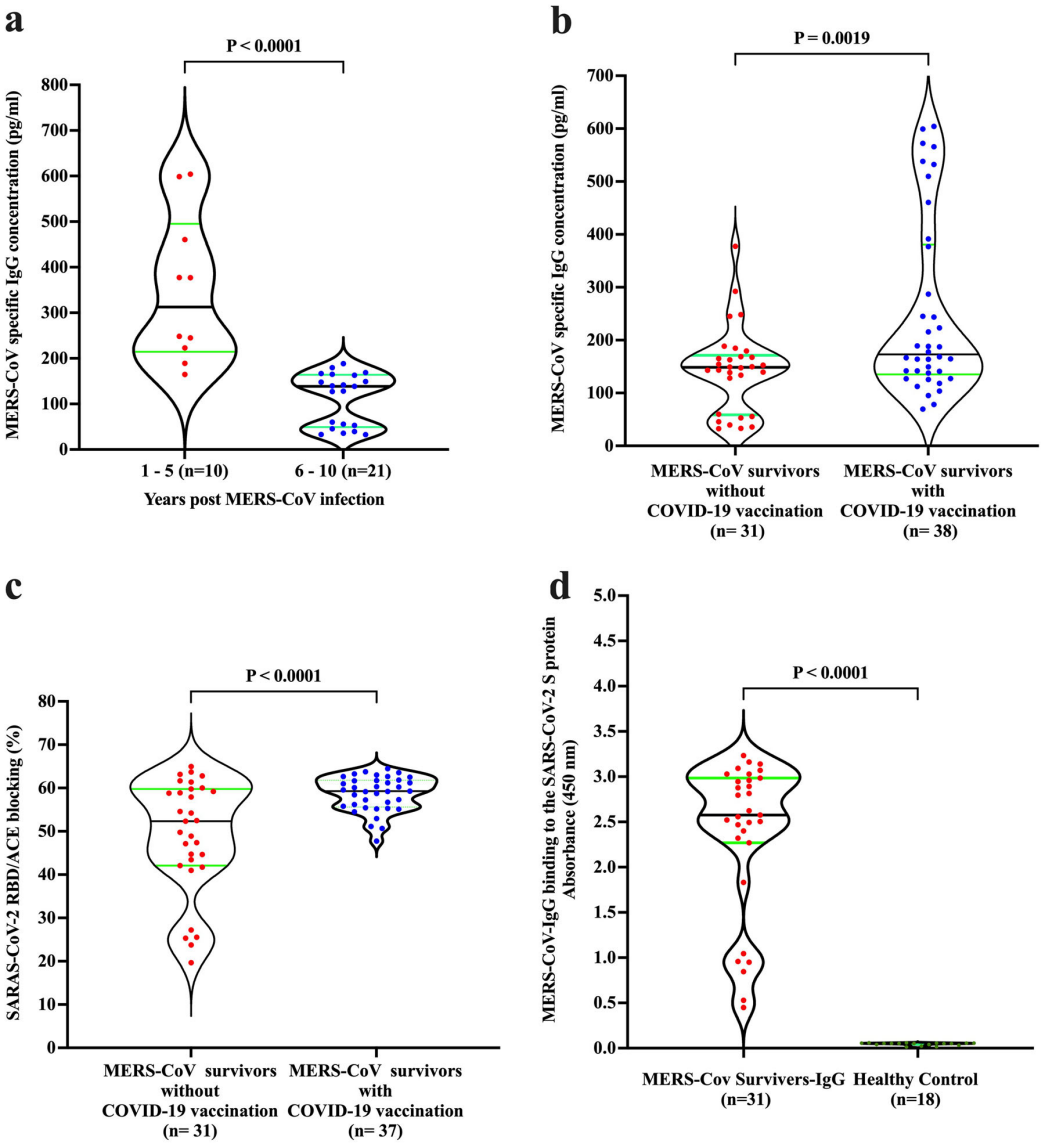
Data on the persistence of MERS‐CoV‐specific IgG concentration, a long‐lasting humoral response and MERS‐CoV‐specific IgG bind and cross‐recognize SARS‐CoV‐2. (a) Data on the persistence of MERS‐CoV spike‐specific IgG concentration (pg/mL). (b) MERS‐CoV spike‐specific IgG antibody levels were measured at 1–5 years and 6–10 years, respectively (pg/mL). (c) Functional blocking of ACE2 binding to SARS‐CoV‐2 RBD, due to limited samples only (*n* = 37) were included. (d) MERS‐CoV‐specific IgG binds and cross‐recognizes SARS‐CoV‐2 spike protein (optical density [OD]). We performed a pilot reverse experiment to measure MERS‐IgG binding to the spike protein of SARS‐CoV‐2 using an ELISA plate coated with SARS‐CoV‐2 spike protein. The results are presented as violin plots, where black lines indicate the median and green lines represent the upper and lower quartiles. Each red and blue dot represents one plasma sample.

All MERS‐CoV‐recovered individuals without COVID‐19 vaccination (*n* = 31) had positive anti‐MERS‐CoV IgG (mean 158.51 ± 111.52 pg/mL) beyond several years postinfection. The levels of anti‐MERS‐CoV IgG were significantly higher in the 1–5 years group postinfection (mean 348.78 ± 154.24 pg/mL) compared to the 6–10 years group after recovery (*p* < 0.0001) (Figure 1a). MERS‐CoV survivors who received 2 doses of COVID‐19 mRNA vaccine exhibited a significantly higher level of specific anti‐MERS‐CoV IgG antibodies (*p* < 0.0019) compared to MERS‐CoV survivors who were not vaccinated against COVID‐19 (Figure 1b). Both MERS‐CoV‐recovered participants with and without the COVID‐19 vaccine showed significantly high levels of MERS‐CoV IgG (Figure 1b).

### 3.2 MERS‐CoV‐Recovered Individuals Demonstrated Functional SARS‐CoV‐2/RBD‐ACE2 Blocking IgG Antibodies

To assess the functionality and cross‐reactivity of persistent MERS‐CoV‐specific IgG antibodies in inhibiting SARS‐CoV‐2. Both MERS‐CoV survivors without COVID‐19 vaccination and those who received 2 doses of COVID‐19 mRNA vaccine demonstrated a high level of inhibitory binding of RBD and ACE2 to wild‐type SARS‐CoV‐2, with a mean inhibitory rate of 48.97% and 58.66%, respectively. However, MERS‐CoV survivors who received two doses of the COVID‐19 mRNA vaccine showed a significantly higher inhibitory rate compared to the non‐vaccinated group (*p* < 0.0001, Figure 1c). In contrast, plasma from healthy unexposed control individuals showed negligible RBD‐ACE2 binding inhibition. These results indicate that prior exposure to MERS‐CoV, with or without vaccination against COVID‐19, is required to generate the RBD‐targeting antibodies capable of blocking the critical RBD‐ACE2 interaction. Based on our results, individuals who had both a previous MERS‐CoV infection and received the COVID‐19 vaccination exhibited a strong spike‐specific antibody response. The combination of previous MERS‐CoV infection and COVID‐19 vaccination may synergistically impact the immune response. When these individuals received the COVID‐19 vaccine, which targets the spike protein of the SARS‐CoV‐2, it likely triggered an enhanced immune response due to existing immune memory. Our findings suggest that individuals with a history of MERS‐CoV infection may benefit from COVID‐19 vaccination in terms of antibody response.

### 3.3 Long‐Lived MERS‐CoV IgG Cross‐Recognized SARS‐CoV‐2

When comparing the plasma of MERS‐CoV survivors without COVID‐19 vaccination to healthy control plasma, we observed significant (*p* < 0.0001) binding to SARS‐CoV‐2 spike proteins (optical density [OD] 2.66 ± 0.83) compared to the healthy control (mean O.D 0.04 ± 0.01) (Figure 1d). All MERS‐CoV survivors without COVID‐19 vaccination exhibited significant levels of spike‐binding IgG.

### 3.4 MERS‐CoV‐Specific CD4^+^ and CD8^+^ T‐Cell Response

**Figure 2:**
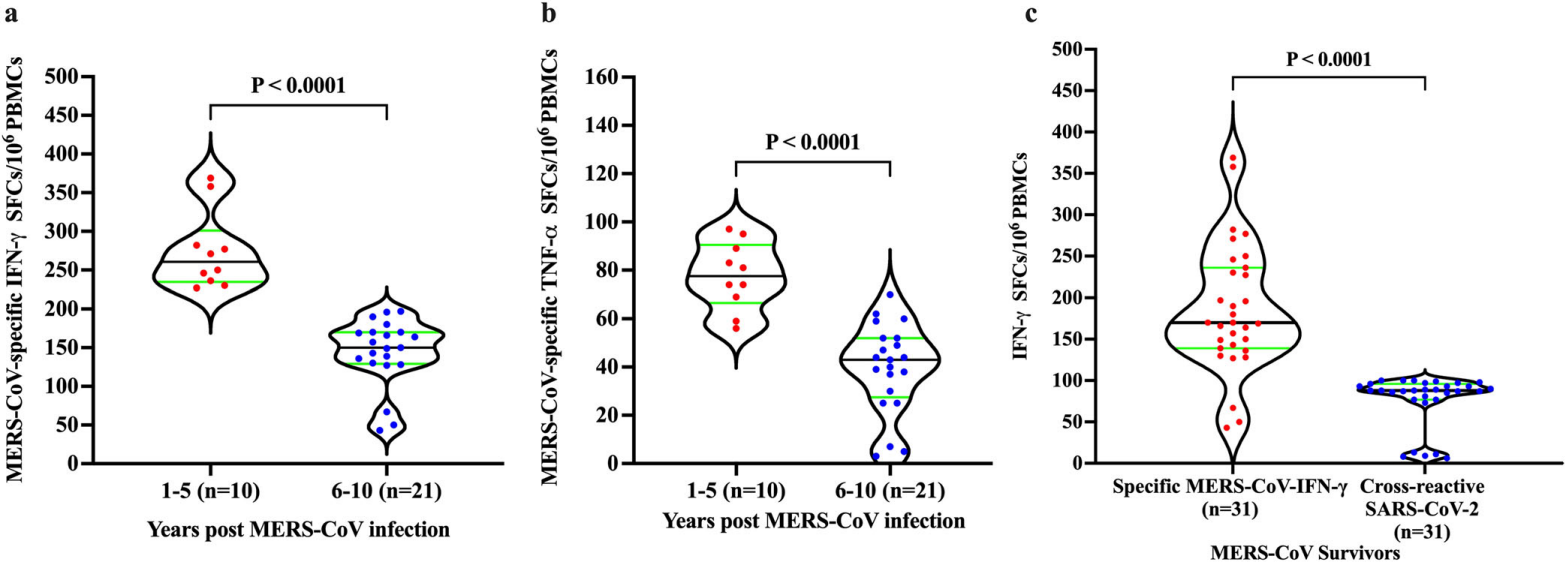
MERS‐CoV‐specific and cross‐reactive SARS‐CoV‐2 T‐cell memory response. (a) The number of IFN‐γ secreting CD8^+^ T cells among 1–5 years and 6–10 years postinfection groups. (b) The number of TNF‐α secreting CD4^+^ T cells among 1–5 years and 6–10 years postinfection groups. (c) Cross‐reactive SARS‐CoV‐2 CD8^+^ T cells producing IFN‐γ. To study cross‐reactive SARS‐CoV‐2 and MERS‐CoV specific T‐cell responses among MERS‐CoV‐recovered individuals without COVID‐19 vaccination (*n* = 31), PBMCs were re‐stimulated with either the structural proteins of four MERS‐CoV (EMC strain) or SARS‐CoV peptide pool, and cultured in a 96‐well ELISpot plate for 20 h. This allowed the detection of CD8^+^ T cells producing IFN‐γ and CD4^+^ T cells producing TNF‐α, measured as Spot‐forming cells (SFCs) using TNF‐α and IFN‐γ ELIspots. All results are presented as violin plots, with black lines indicating the median and green lines representing the upper and lower quartiles. Each red and blue dot represents one sample.

MERS‐CoV‐recovered patients demonstrated specific memory T‐cell responses (Figure 2a,b), indicating the presence of a long‐lasting MERS‐CoV‐specific T‐cell response. MERS‐CoV specific CD8^+^ T cell response ranged from 34 to 358 per 1 × 10^6^ PBMCs. In contrast, the TNF‐α‐ spot‐forming cells (SFCs) CD4^+^ T‐cell count was relatively low, ranging from 3 to 97 per 1 × 10^6^ PBMCs. The number of IFN‐γ‐producing CD8^+^ T cells and TNF‐α‐producing CD4^+^ T cells was significantly higher in the 1–5 years group after infection, compared to the 6–10 years group after recovery (*p* = < 0.0001) (Figure 2a,b). A significant finding from our study was the detection of SARS‐CoV‐2‐reactive CD8^+^ T cells in a significant proportion of MERS‐CoV‐recovered participants (Figure 2c). This result suggests the possibility of a cross‐reactivity or immune response between these two coronaviruses.

### 3.5 Intracellular Cytokine Staining Assay (ICS)

**Figure 3:**
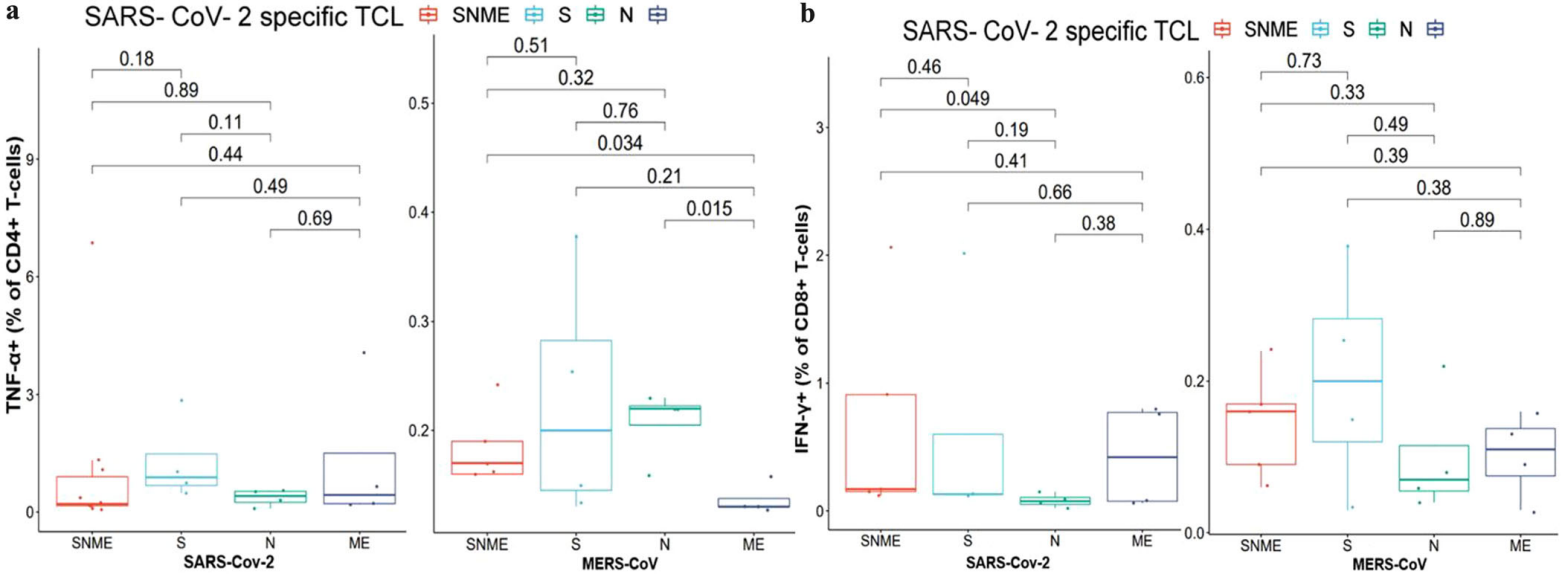
Flow cytometry analysis of SARS‐CoV‐2 T‐cell lines (SARS‐CoV‐2‐TCL). (a) Percentage of TNF‐α positive memory CD4^+^ T cells specific to SARS‐CoV‐2 that cross‐reacted with MERS‐CoV. (b) Percentage of IFN‐γ positive memory CD8^+^ T cells specific to SARS‐CoV‐2 that cross‐reacted with MERS‐CoV.

**Figure 4:**
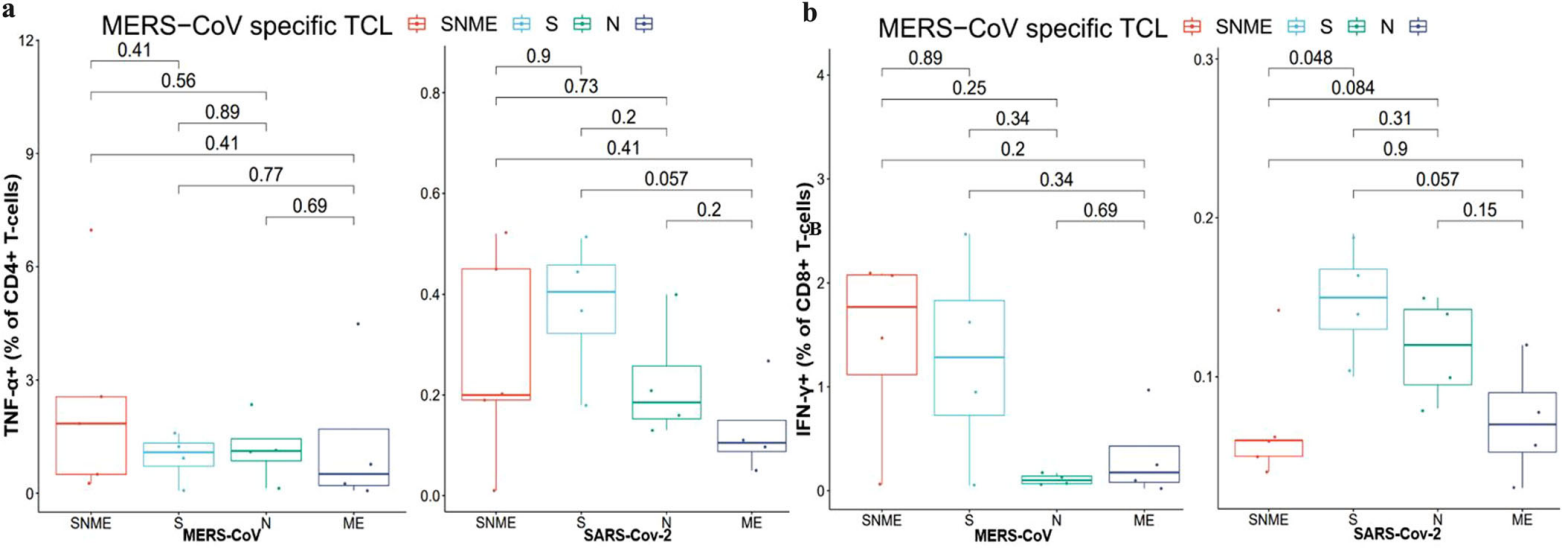
Flow cytometry analysis of MERS‐CoV T‐cell lines (MERS‐CoV‐TCL). (a) Percentage of TNF‐α positive memory CD4^+^ T cells specific to MERS‐CoV that cross‐reacted with SARS‐CoV‐2. (b) Percentage of IFN‐γ positive memory CD8^+^ T cells specific to MERS‐CoV that cross‐reacted with SARS‐CoV‐2.

**Figure 5:**
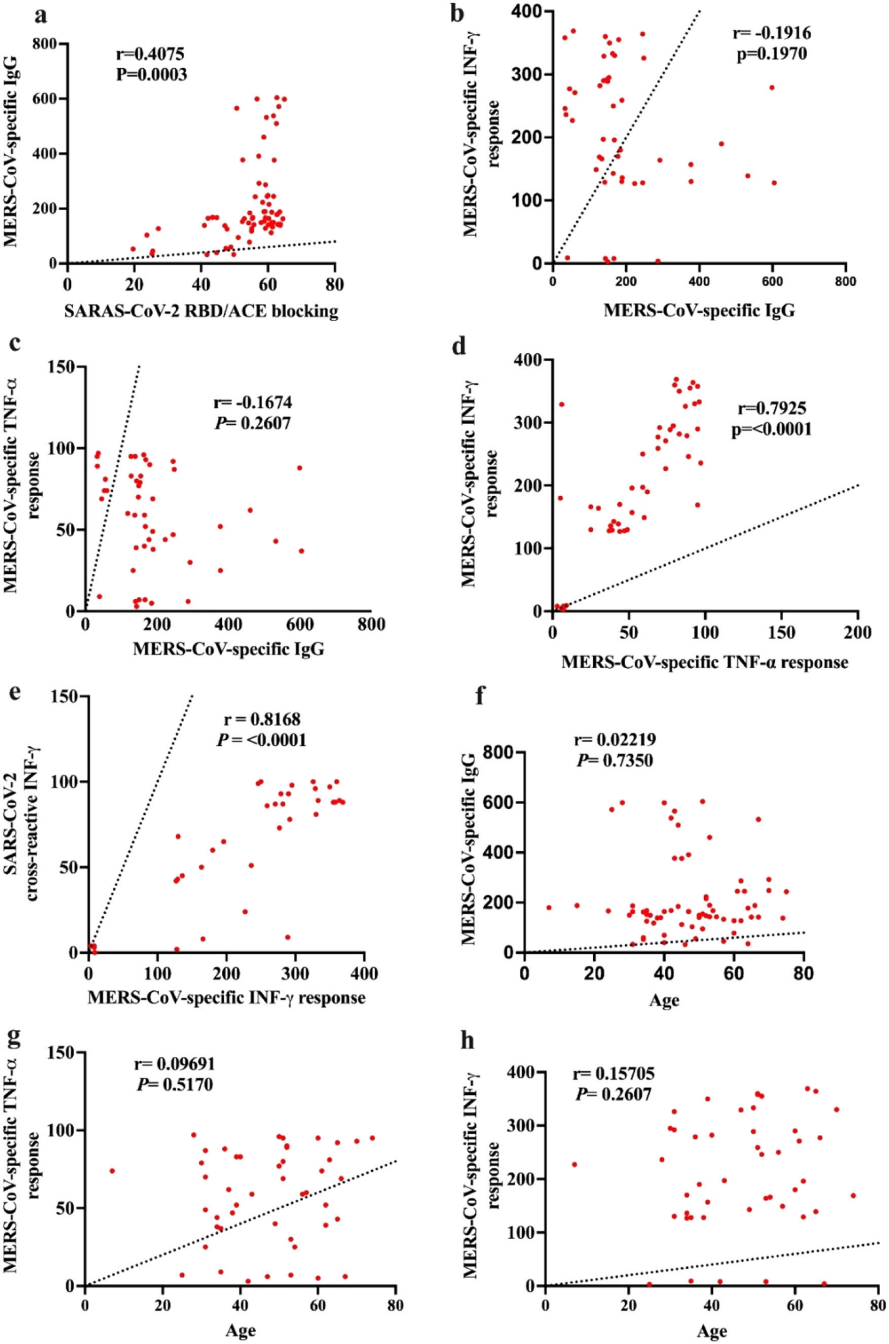
Correlations between MERS‐CoV‐specific IgG, CD8^+^ T cells producing IFN‐γ and CD4^+^ T cells producing TNF‐α and ACE2 binding to SARS‐CoV‐2 RBD. (a) Correlation between MERS‐CoV spike‐specific IgG and SARS‐CoV‐2 RBD/ACE blocking. (b) Correlation between MERS‐CoV‐specific IFN‐γ and MERS‐CoV spike‐specific IgG. (c) Correlation between MERS‐CoV‐specific TNF‐α and MERS‐CoV spike‐specific IgG. (d) Correlation between MERS‐CoV‐specific IFN‐γ and MERS‐CoV‐specific TNF‐α. (e) Correlation between SARS‐CoV‐2 cross‐reactive IFN‐γ and MERS‐CoV‐specific IFN‐γ. (f) Correlation between MERS‐CoV spike‐specific IgG and age. (g) Correlation between MERS‐CoV‐specific TNF‐α and age. (h) Correlation between MERS‐CoV‐specific IFN‐γ and age.

We used ICS to detect specific T‐cell responses. Our results showed detectable CD4^+^ and CD8^+^ T cell responses that are specific to MERS‐CoV and cross‐reactive to SARS‐CoV‐2 (Figure 3) and (Figures 1–5) in the Supporting Information S1.

### 3.6 Short‐Term T‐Cell Line Development and Specific and Cross‐Reactive T‐Cell Detection

The TCLs developed with peptide pools from the the N‐ and C‐terminal portions of the spike (S1 and S2) glycoprotein, the nucleocapsid (N) protein and the transmembrane with the envelope (ME) protein of SARS‐CoV‐2 (SNME) (SARS‐CoV‐2‐TCL) showed the presence of SARS‐CoV‐2 specific T cells that cross‐reacted with MERS‐CoV. These cells were detected in all participants and were able to recognize spike proteins of both MERS‐CoV and SARS‐CoV‐2 (Figures 3 and 4) and (Supporting Information S1: Figures 2–5). Additionally, the TCLs developed with peptide pools from the the N‐ and C‐terminal portions of the spike (S1 and S2) glycoprotein, the nucleocapsid (N) protein and the transmembrane with the envelope (ME) protein of MERS‐CoV proteins (SNME) (MERS‐TCL) revealed the presence of MERS‐CoV specific CD4^+^ T cells that cross‐reacted with SARS‐CoV‐2. These cells also recognized spike proteins of both MERS‐CoV and SARS‐CoV‐2 (Figures 3 and 4). Specific and cross‐reactive T‐cell responses in TCLs toward SARS‐CoV‐2 and MERS‐CoV highlighted the potential cross‐reactivity between SARS‐CoV‐2 and MERS‐CoV. It is important to note that we were only stimulating PT1 TCL with the SARS‐CoV‐2 (SNME) or MERS‐CoV (SNME) pools (Figures 3 and 4). Since limited PT1 PBMC cells were recovered, the proliferated TCL cells were insufficient for stimulation with separated peptide pools. PT6,7,8, and 9 had enough PBMC cells to develop TCL cells.

### 3.7 Correlation Between MERS‐CoV‐Spike‐Specific IgG, SARS‐CoV‐2 RBD Binding ACE2, IFN‐γ‐Producing Memory CD8^+^ T Cells TNF‐α Producing Memory CD4^+^ T Cell Responses

Pearson's correlation analysis was used to investigate the relationship between MERS‐CoV‐spike‐specific IgG, ACE2 binding to SARS‐CoV‐2 RBD (%), IFN‐γ‐producing memory CD8^+^ T cells, and TNF‐α‐producing memory CD4^+^ T cell responses. The analysis revealed a positive and significant correlation between MERS‐CoV IgG levels in all participants and SARS‐CoV‐2 RBD/ACE blocking (*r* = 0.4075; *p* = 0.0003) (Figure 5a). After 1–5 and 6–10 years postinfection, MERS‐CoV IgG antibody levels were significantly correlated with inhibition and blocking activity against SARS‐CoV‐2. The IFN‐γ‐producing memory CD8^+^ T cell response showed a significant correlation with the TNF‐α‐producing memory CD4^+^ T cell response (*r* = 0.7925; *p*< 0.0001) (Figure 5d). Additionally, a significant correlation (*r* = 0.8168; *p* < 0.0001) was found between MERS‐CoV‐specific IFN‐γ‐memory CD8^+^ T cell responses and SARS‐CoV‐2 cross‐reactive IFN‐γ (Figure 5e). The memory CD8^+^ T cells producing IFN‐γ and memory CD4^+^ T cells producing TNF‐α did not show a correlation with anti‐MERS‐CoV IgG levels (Figure 5b,c). We also investigated the impact of age on the memory T‐cell and humoral responses in MERS‐CoV patients who had recovered. There was no correlation between TNF‐α‐producing memory T‐cell responses and age (Figure 5g). We found no significant correlation between IFN‐γ levels and MERS‐CoV‐specific IgG levels with age (Figure 5f,h).

### 3.8 Focus Reduction Neutralization Test

**Figure 6:**
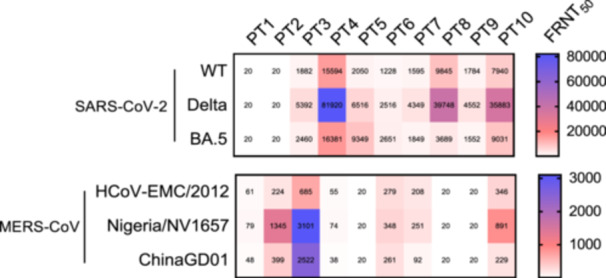
Neutralization titer of MERS‐CoV strains (HCoV‐EMC/2012, ChinaGD01, and Nigeria/NV1657) and SARS‐CoV‐2 strains (Wild type [WT], Delta variant, and BA.5 variant). Only 10 participants' samples were assayed (P1–P10 indicates participant numbers). FRNT 50 could not be performed for all participants due to the limited sample volume.

The neutralizing activity was assessed using focus reduction neutralization test (FRNT) 50% values ranging from less than 20 to greater than 3000. The SARS‐CoV‐2 variants, including the wild type (WT), Delta variant, and BA.5 variant displayed significant neutralizing activity. The results demonstrate the variation in neutralization titers among different strains of MERS‐CoV, SARS‐CoV‐2, and its variants (Figure 6). FRNT 50% was conducted, for only 10 participants. The combination of previous MERS‐CoV infection and COVID‐19 vaccination may synergistically impact the immune response.

## 4 Discussion

We examined the longevity of humoral and T‐cell responses to MERS‐CoV, including neutralizing antibodies from MERS‐CoV recovered individuals against different MERS‐CoV isolates (HCoV‐EMC/2012, Nigeria/NV1657, ChinaGD01), as well as SARS‐CoV‐2 variants (WT, Delta, and BA.5). Our study shows that individuals recovered from MERS‐CoV have long‐lasting humoral and cellular immune responses. We found specific IgG levels against MERS‐CoV and its neutralizing ability against MERS‐CoV and SARS‐CoV‐2 variants (WT, Delta, BA.5). Those previously infected with MERS‐CoV and vaccinated for COVID‐19 showed strong immune responses. This suggests additional benefits from COVID‐19 vaccination for those with a history of MERS‐CoV infection. To the best of our knowledge, this is the first study to demonstrate that IgG antibodies against MERS‐CoV can persist for up to 10 years. Previous studies indicated that individuals infected with MERS‐CoV exhibit long‐lasting virus‐specific immune responses up to 6.9 years after the initial infection [2]. Similar observations have been made in individuals who recovered from SARS‐CoV‐1 infection, with studies reporting the persistence of T‐cell and antibody responses over many years [19, 27, 28]. However, it is important to note that the evaluation of antibody responses in those studies only reflects the persistence of immunity. In contrast, our findings provide insights into both humoral and T‐cell responses, including neutralizing antibodies against different MERS‐CoV isolates, as well as the Wild‐type and variants of SARS‐CoV‐2. Two studies have also shown antibody cross‐reactivity between MERS‐CoV and SARS‐CoV‐2 [29, 30]. Our results provide direct evidence of the presence of cross‐reactive neutralizing antibodies to SARS‐CoV‐2 from MERS‐CoV‐survived individuals [14, 15, 25]. The genetic and antigenic relationships between MERS‐CoV, SARS‐CoV‐1, and SARS‐CoV‐2 provide a potential mechanistic basis for this cross‐reactivity. As members of the same Betacoronavirus genus, these viruses share significant sequence homology, particularly in the spike protein receptor‐binding domain (RBD) that is a key target of neutralizing antibodies [31, 32]. Prior work has shown that MERS‐CoV RBD has around 50% amino acid identity with SARS‐CoV‐2 RBD, which likely enables some degree of epitope cross‐recognition [29, 32]. In this study, we showed that the long‐lasting MERS‐CoV‐specific IgG is still functional, and bound and inhibits the interaction between the SARS‐CoV‐2 RBD protein and the ACE2 receptor. This inhibitory effect persists up to ten years after infection. Several studies have shown that SARS‐CoV‐1 is closely related to MERS‐CoV and other alpha CoVs [15, 33]. Additionally, it has been found that serum from convalescent SARS‐1 patients or animals can cross‐neutralize SARS‐CoV‐2 by blocking the interaction between SARS‐CoV‐2 and ACE2 [30, 34].

In this study, we found that MERS‐CoV survivors who received two doses of the COVID‐19 vaccine exhibited significantly higher levels of specific anti‐MERS‐CoV IgG antibodies compared to MERS‐CoV survivors who were not vaccinated against COVID‐19. Additionally, both MERS‐CoV survivors without COVID‐19 vaccination and those who received two doses of the COVID‐19 vaccine demonstrated a high level of inhibitory binding of the RBD and ACE2 to wild‐type SARS‐CoV‐2. However, MERS‐CoV survivors who received two doses of the COVID‐19 vaccine showed a significantly higher inhibitory rate compared to the non‐vaccinated group. These findings suggest that the combination of previous MERS‐CoV infection and COVID‐19 vaccination may have a synergistic effect on the immune response [35, 36]. Our results showed that the MERS‐CoV strains HCoV‐EMC/2012, Nigeria/NV1657, and ChinaGD01 showed neutralizing activity. Wild‐type SARS‐CoV‐2, including its variants Delta variant and BA.5 variant, also exhibited significant neutralizing activity. A retrospective cohort study specifically focused on individuals who previously had MERS‐CoV infection suggested that they exhibit a cross‐reactive immune response, providing some level of protection against SARS‐CoV‐2 [30]. However, the same study also points out that these individuals had higher risks of COVID‐19‐related hospitalization and death than MERS‐CoV‐negative individuals. Another study shows that patients with previous MERS‐CoV infection who received the COVID‐19 vaccine demonstrated a significant boost in cross‐reactive, neutralizing antibodies [37]. The presence of pre‐existing immunity due to previous MERS‐CoV infection combined with COVID‐19 vaccination may have an impact on the immune response to subsequent SARS‐CoV‐2 infection or vaccination.

Previous studies have shown cellular immune memory, generated from either primary infection or vaccination, plays a critical role in protection from re‐infection [38, 39]. The present study corroborates other literature, demonstrating MERS‐CoV‐specific long‐lasting memory CD4^+^ and CD8^+^ T cell responses regardless of disease severity. There is a significant difference in memory CD4^+^ and CD8^+^ T cell responses between two groups (1–5 and 6–10 years) postinfection [2, 13]. Our findings are consistent with a recent study that found MERS‐CoV‐specific memory CD8^+^ and CD4^+^ T cells persisted in the body for 6.9 years after recovery [2]. Previous studies reported that SARS‐CoV‐1 membrane and nucleocapsid proteins induced two memory CD8^+^ T cell responses, which can persist for more than 10 years postinfection [21]. These findings could be attributed to the abundant MERS‐CoV spike protein antigen presentation through MHC class 1 during infection, which results in a higher number of specific CD8^+^ T cells. Cross‐reactive T cells between SARS‐CoV‐2 and other common cold coronaviruses, such as NL63 and OC43, have been reported [40, 41, 42].

It is important to note that none of the participants in this study had a history of re‐infection. Although MERS‐CoV cases are still being reported in the study country. Our results indicated a significant correlation between MERS‐CoV IgG antibody levels and inhibition/blocking activity against SARS‐CoV‐2. Furthermore, we observed a notable association between IFN‐γ‐producing memory CD8^+^ T cells and TNF‐α‐producing memory CD4^+^ T cells in both vaccinated and non‐vaccinated participants. On the other hand, there was no correlation between memory T‐cell responses and age. One possible reason for this lack of correlation is that the sample size or age distribution of the participants was insufficient to detect any meaningful age‐related differences. Previous studies have shown that age can impair and dysregulate the host immune response during SARS‐CoV‐2 infection [43, 44]. The remarkable durability of the cellular and humoral immune responses elicited by MERS‐CoV infection, and the cross‐reactivity of MERS‐CoV‐specific memory T cells and antibodies with the wild‐type SARS‐CoV‐2 and its variants, demonstrated in this study, may have significant implications for the development of a pan‐coronavirus vaccine. The cross‐reactivity observed between MERS‐CoV and SARS‐CoV‐2 immunity is a significant finding. Our analysis of both T cell and antibody responses revealed substantial cross‐recognition, likely driven by the conservation of critical epitopes across the viral proteins. The ability of MERS‐CoV‐induced immune responses to neutralize SARS‐CoV‐2 suggests potential “one‐way” cross‐protection, where prior exposure to MERS‐CoV may confer some level of protection against the wild‐type SARS‐CoV‐2 strain and its variants. Furthermore, the identification of shared epitopes targeted by MERS‐CoV and SARS‐CoV‐2 immune responses could inform the design of universal or pan‐coronavirus vaccines capable of eliciting broad protection.

## 5 Conclusions

Our study provides valuable information on the long‐lasting humoral and cellular immune responses in individuals who have recovered from MERS‐CoV. We demonstrated the presence of specific IgG levels against MERS‐CoV, as well as its ability to neutralize MERS‐CoV (HCoV‐EMC/2012, Nigeria/NV1657 and ChinaGD01) and SARS‐CoV‐2 variants (WT, Delta, and BA.5). We found that individuals who had previously been infected with MERS‐CoV and received the COVID‐19 vaccination exhibited a strong humoral and cellular immune response. Our findings suggest that individuals with a history of MERS‐CoV infection may experience additional benefits from COVID‐19 vaccination regarding antibody and T‐cell response. This indicates that the COVID‐19 vaccination was able to boost and reshape the long‐term humoral immune memory established from the original MERS‐CoV infection. Given the rapidly evolving landscape of SARS‐CoV‐2, evaluating the cross‐reactivity of MERS‐CoV immunity against the latest Omicron subvariants is important. This manuscript presents data on the immune responses of MERS‐CoV survivors to the original SARS‐CoV‐2 strain and early variants such as Delta and BA.5. However, the emergence of highly immune‐evasive Omicron subvariants raises the question of whether MERS‐CoV‐elicited immune responses can also cross‐react with these newer variants. We recommend future studies to assess the neutralization capacity of MERS‐CoV survivor sera against predominant Omicron subvariants.

Our study had several limitations that need to be acknowledged. First, the proliferation of T‐cell lines was conducted using only a small number of individual PBMC cells. Second, the plasma of recovered MERS‐CoV patients may not accurately reflect the host response to viral infection in the airways. It is crucial to consider these limitations when interpreting the results of the current study.

## Author Contributions

*Study design:* Bandar Alosaimi, Maaweya Awadalla, Wael Alturaiki, and Haitham S. Alkadi. *Performed the analyses:* Zhao Chen, Zhaoyong Zhang, Airu Zhu, Fatimah Rebh, and Abeer N. Alshukairi. *Data collection and study management:* Bandar Alosaimi, Maaweya Awadalla, Abeer N. Alshukairi, and Haitham S. Alkadi. *Interpretation of results:* Jincun Zhao, Zhao Chen, Zhaoyong Zhang, Airu Zhu, Bandar Alosaimi, Maaweya Awadalla, and Haitham S. Alkadi. Bandar Alosaimi, Maaweya Awadalla, and Wael Alturaiki wrote the manuscript draft, which was reviewed and revised by all authors.

## Conflicts of Interest

The authors declare no conflicts of interest.

## Supporting information

Supporting information.

Supporting information.

## Data Availability

The data that support the findings of this study are available from the corresponding author upon reasonable request.
