# Supplementary material for: A 10 Year Long‐Lived Cellular and Humoral MERS‐CoV Immunity Cross‐Recognizing the Wild‐Type and Variants of SARS‐CoV‐2: A Potential One‐Way MERS‐CoV Cross‐Protection Toward a Pan‐Coronavirus Vaccine
Source: J Med Virol. 2025 Jan 17;97(1):e70071. doi: 10.1002/jmv.70071 (PMC11740004; doi:10.1002/jmv.70071)
Supplement: Supplementary file 1 — Supporting information. [file JMV-97-e70071-s001.docx]

**A 10 year long-lived cellular and humoral MERS-CoV immunity cross-recognizing the Wild-type and Variants of SARS-CoV-2: a potential one-way MERS-CoV cross-protection towards a pan-coronavirus vaccine**

Bandar Alosaimi^1,7^; Maaweya Awadalla^1,*, 7^; Wael Alturaiki^2^; Zhao Chen^3^; Zhaoyong Zhang^3^; Airu Zhu^3^; Fatimah Rebh^4^; Abeer N Alshukairi^5^; Jincun Zhao^3,6^; Haitham S. Alkadi.^1^


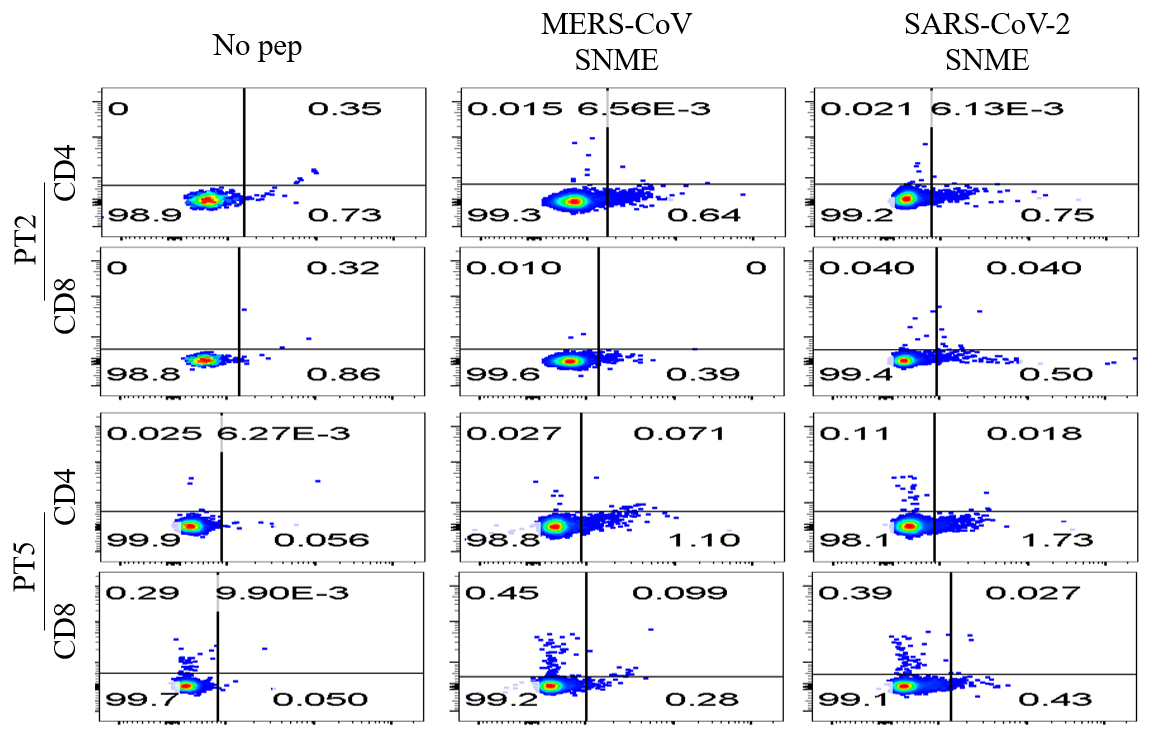


**IFN-γ**

**TNF-α**

**Figure S1. ICS and flow cytometry analysis of restimulated PBMCs from MERS-CoV survivors.** Representative ICS flow cytometry plots were analyzed to identify CD4^+^ and CD8^+^ T cells expressing IFN-γ (x-axis) and TNF-α (y-axis) in response to peptide pools from the S, N, and ME regions. We used combined SNME peptide pools derived from the N- and C-terminal portions of the spike (S1 and S2) glycoprotein, the nucleocapsid (N) protein, and the transmembrane with the envelope (ME) protein of MERS-CoV (EMC strain) or SARS-CoV-2 (WT strain) for PBMC stimulation. The numbers within the gates indicate the percentages of positive cells. Specific CD4^+^ and CD8^+^ T cell responses against MERS-CoV and SARS-CoV-2 were detectable. However, in participant (PT2), no specific CD4^+^ and CD8^+^ T cell responses were observed.


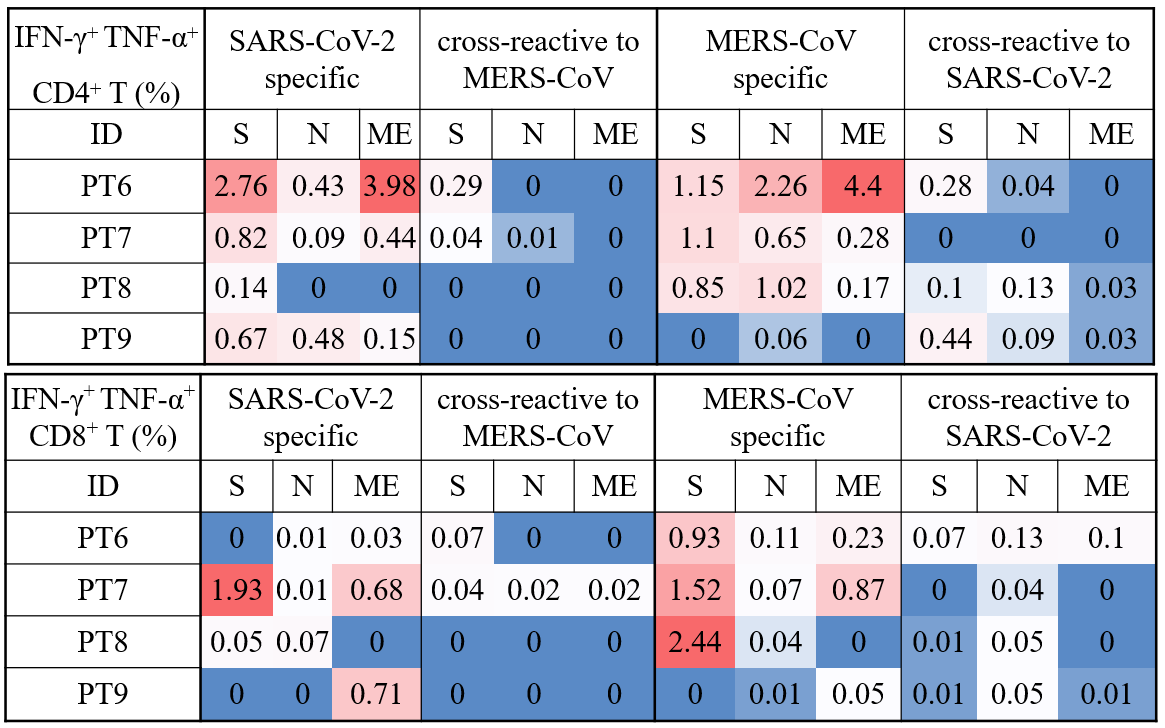


**Figure S2. Specific and cross-reactive T-cell responses in TCLs (T-cell lines) towards two SARS-CoV-2 and MERS-CoV.** A comprehensive summary of the specific and cross-reactive T-cell responses in TCLs (T-cell lines) towards MERS-CoV and SARS-CoV-2. In addition to combined "SNME" peptide pools, we used individual S, N, M, and E peptide pools of MERS-CoV (EMC strain) or SARS-CoV-2 (WT strain) for PBMC stimulation. The numbers in the table are subtractions of percentages. The red and blue cells indicate the percentage rate of specific and/or CD4+ and CD8+ T cell cross-reactivity between MERS-CoV and SARS-CoV-2. The figure presents and highlights the potential cross-reactivity between MERS-CoV and SARS-CoV-2.

MERS-CoV

specific IFN-γ


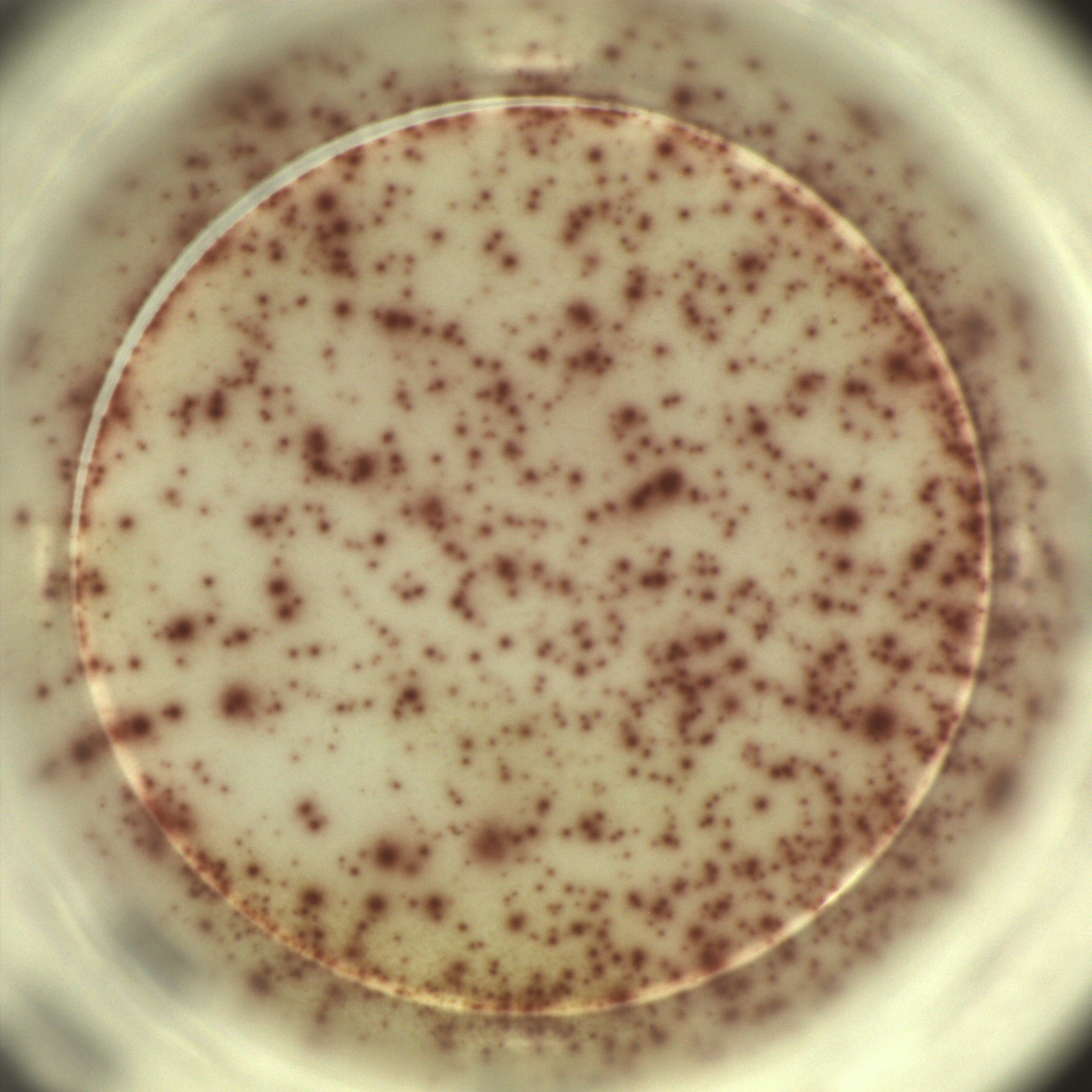


MERS-CoV

specific TNF-α


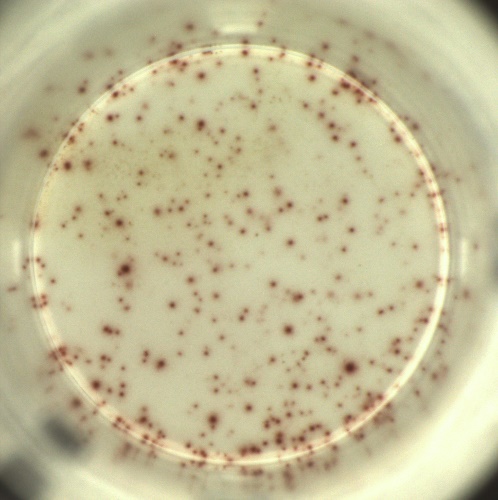


Positive control


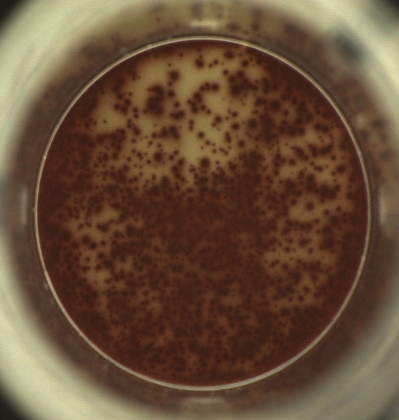


Negative control


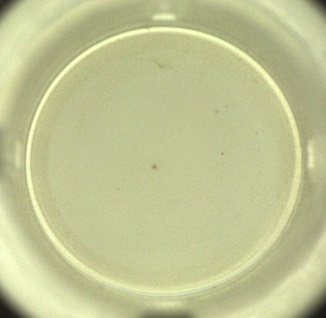


**Figure S3.** Representative CD8+ T cells producing IFN-γ and CD4+ T cells producing TNF-α ELISpot images, positive control, and negative control wells of PBMCs.


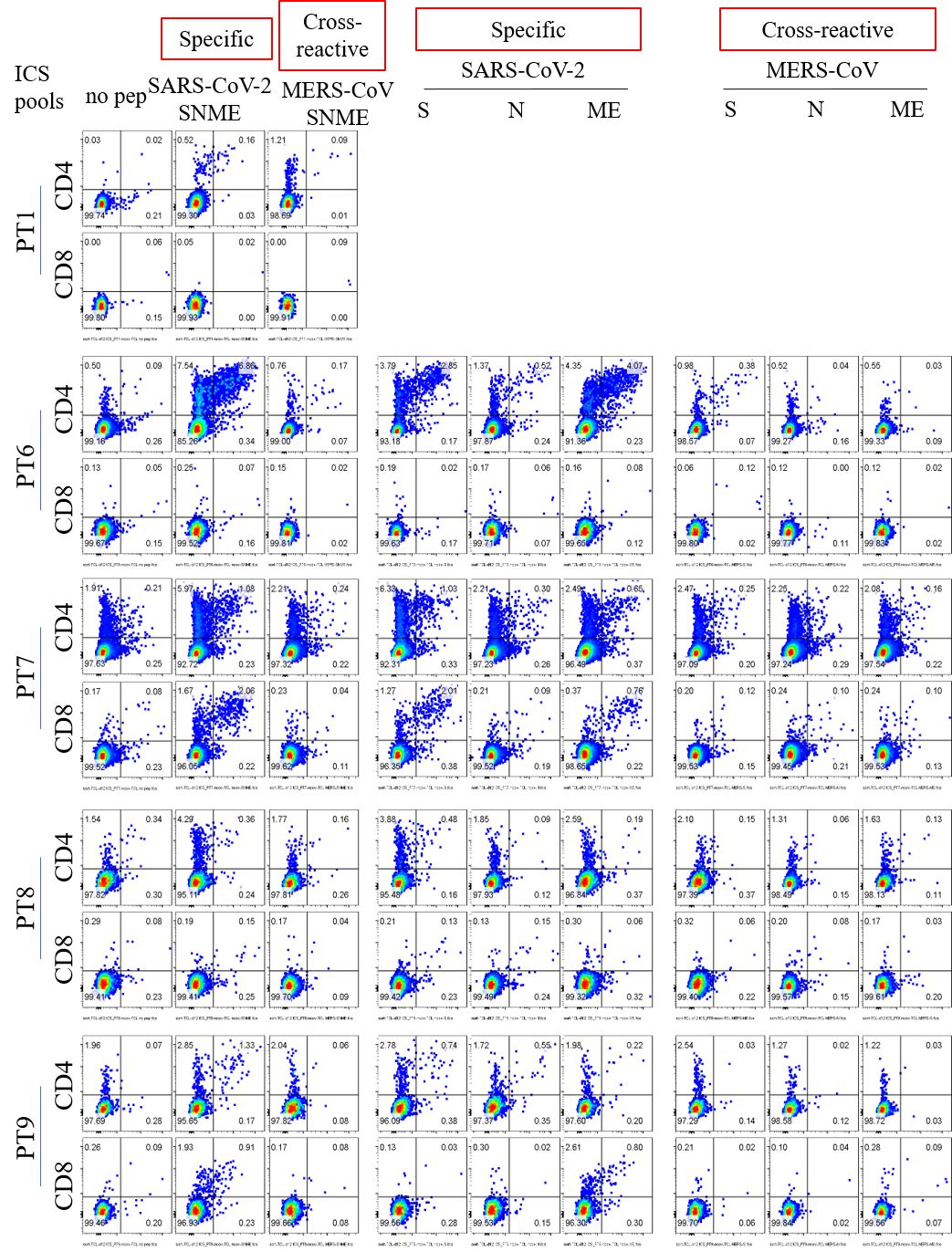


**TNF-α**

**IFN-γ**

**Figure S4. Flow cytometry analysis of SARS-CoV-2 T-cell lines (SARS-CoV-2-TCL).**

ICS assays used peptide pools from the S1, S2, N, and ME regions (SNME). SNME regions included the N- and C-terminal portions of the spike (S1 and S2) glycoprotein, the nucleocapsid (N) protein, and the transmembrane with the envelope (ME) protein of MERS-CoV (EMC strain) and SARS-Cov-2 (WT strain). The assays were performed on TCLs developed with SARS-CoV-2 proteins (SARS-CoV-2-TCL) to evaluate specific and cross-reactive T cell responses towards SARS-CoV-2 and MERS-CoV. The x-axis represents IFN-γ, while the y-axis represents TNF-α. SARS-CoV-2 specific CD4+ T cells that cross-reacted to MERS-CoV were detected. (P1, P6, P7, P8 and P9 indicate participant numbers). We stimulated PT1 TCL with the whole SARS-CoV-2 (SNME) or MERS-CoV (SNME) pools. Since limited PT1 PBMC cells were recovered, the proliferated TCL cells were insufficient for stimulation with separated peptide pools. The numbers in the gates indicate the percentages of positive cells. No pep refers to the absence of stimulation with peptides, serving as a control in our experiments.


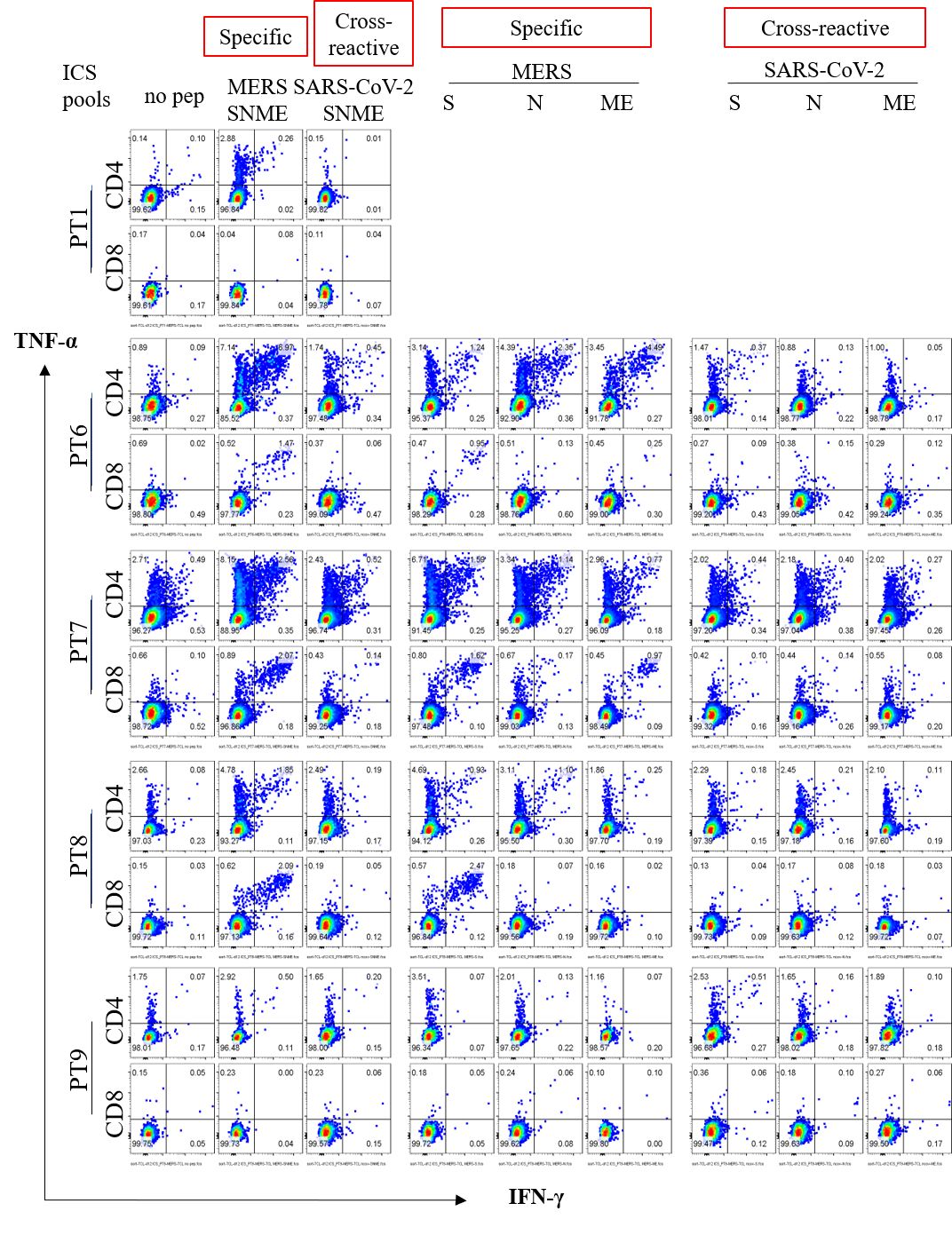


**Figure S5. Flow cytometry analysis of MERS-CoV T-cell lines (MERS-CoV-TCL).**

ICS assays used peptide pools from the S1, S2, N, and ME regions (SNME). SNME regions included the N- and C-terminal portions of the spike (S1 and S2) glycoprotein, the nucleocapsid (N) protein, and the transmembrane with the envelope (ME) protein of MERS-CoV (EMC strain) and SARS-Cov-2 (WT strain). The assays were performed on TCLs developed with MERS-CoV (SNME) proteins (MERS-CoV-TCL) to evaluate specific and cross-reactive T cell responses towards MERS-CoV and SARS-CoV-2. The x-axis represents IFN-γ, while the y-axis represents TNF-α. MERS-CoV-specific CD4+ T cells that cross-reacted to SARS-CoV-2 were detected. (P1, P6, P7, P8 and P9 indicate participant numbers). We stimulated PT1 TCL with the whole SARS-CoV-2 (SNME) or MERS-CoV (SNME) pools. Since limited PT1 PBMC cells were recovered, the proliferated TCL cells were insufficient for stimulation toward separated peptide pools. The numbers in the gates indicate the percentages of positive cells. No pep refers to the absence of stimulation with peptides, serving as a control in our experiments.
